# Mechanical, Dielectric and Flame-Retardant Properties of GF/PP Modified with Different Flame Retardants

**DOI:** 10.3390/polym16121681

**Published:** 2024-06-13

**Authors:** Jingwen Li, Yiliang Sun, Boming Zhang, Guocheng Qi

**Affiliations:** 1 School of Materials Science and Engineering, Beihang University, Beijing 100191, China; ljw666@buaa.edu.cn (J.L.); zbm@buaa.edu.cn (B.Z.); 2 Department of Mechanics, Beijing Jiaotong University, Beijing 100044, China; gchqi@bjtu.edu.cn

**Keywords:** flame retardant-modified glass fiber-reinforced polypropylene, mechanical properties, dielectric properties, flame-retardant properties, gray relational analysis

## Abstract

With the rapid development of electronic information technology, higher requirements have been put forward for the dielectric properties and load-bearing capacity of materials. In continuous glass fiber-reinforced thermoplastic composites, polypropylene matrix is a non-polar polymer with a very low dielectric constant and dielectric loss, but polypropylene is extremely flammable which greatly limits its application. Aiming at the better application of flame retardant-modified continuous glass fiber-reinforced polypropylene composites (FR/GF/PP) in the field of electronic communication, the effects of four different kinds of flame retardants (Decabromodiphenyl ethane (DBDPE), halogen-free one-component flame retardant (MONO), halogen-free compound flame retardant (MULTI), and intumescent flame retardant (IFR)) on the properties of FR/GF/PP were compared, including the mechanical properties, dielectric properties and flame-retardant properties. The results showed that among the FR/GF/PP, IFR has the highest performance in mechanical properties, MULTI has better performance in LOI, DBDPE and IFR have better performance in flame retardant rating, and DBDPE and IFR have lower dielectric properties. Finally, gray relational analysis is applied to propose an approach for selecting the optimal combination (flame retardant type and flame-retardant content) of comprehensive performance. In the application exemplified in this paper, the performance of IFR-3-modified GF/PP is optimized.

## 1. Introduction

With the rapid development of electronic information technology, the technical strength and accuracy of antenna systems [1,2] are becoming higher, the frequency of electromagnetic wave transmission and reception is becoming higher, and the signal transmission is becoming faster [3,4,5]. This imposes higher requirements on the wave-transparent properties of materials. For wave-transparent materials, the larger the dielectric constant or the thicker the material, the greater the reflection of electromagnetic waves, and the signal transmission efficiency will be reduced [6,7,8]. The larger the dielectric loss tangent tan δ is, the more energy is lost, as the electromagnetic wave energy is converted to heat in the process of transmitting through the material [9,10]. Therefore, it is required that the thickness of the wave-transmitting material is as small as possible, the dielectric constant is as low as possible, and the loss angle tangent is as low as close to zero in order to minimize reflection and maximize transmission. This places high demands on the dielectric and mechanical properties of the materials [11].

Thermoplastic composites, as a major branch of composites, have significant advantages such as rapid prototyping, being repairable, having secondary molding, and being recyclable and environmentally friendly, in addition to the advantages of light weight and high strength [12,13,14]. Continuous glass fiber (GF) reinforced thermoplastic composites with excellent performance and a low price are widely used in aerospace, building materials, vehicles and other fields. The polypropylene (PP) matrix is a non-polar polymer with a very low dielectric constant and dielectric loss, and its dielectric properties remain stable under variable temperature and frequency, while polypropylene has low water absorption (water absorption increases the dielectric properties) [15,16,17,18]. Polypropylene’s excellent wave-transparent properties make it ideally suited in wave-transparent materials. However, PP has poor flame retardancy, which greatly limits its application [19,20,21]. Continuous fiber reinforced polypropylene composites (GF/PP) with high mechanical properties, good flame-retardant properties, low dielectric constant and low dielectric loss are beneficial to its application in wave-transparent materials. At present, the research on the properties of FR/GF/PP mostly stays in the study of flame-retardant properties and mechanical properties [22,23,24,25], and there is less research on dielectric properties. In order to utilize the FR/GF/PP for better applications in the field of electronic communication, such as in radomes, cabinet casings, plastic vibrators, reflector plates, filters, etc., more studies are needed to be carried out on the dielectric properties of the FR/GF/PP. In previous studies [26], it was found that a large amount of flame-retardant addition would lead to an increase in the dielectric constant and dielectric loss of GF/PP, but there are many types of flame retardants and the effects of different types of flame retardants on the performance of FR/GF/PP may be different, so the question of how to choose the flame retardants needs to be solved urgently.

In this paper, four types of flame retardants which are relatively common in engineering applications were selected. The effects of the types and contents of these four flame retardants on the mechanical, dielectric and flame-retardant properties of FR/GF/PP were compared. Finally, the gray relational analysis [27,28,29] was applied to transform the multi-objective, multi-response problem into a single gray relational comparison problem in order to obtain the flame retardant type and the content that would make the comprehensive performance of FR/GF/PP optimal. It provides a reference for the practical application of FR/GF/PP.

## 2. Materials and Methods

### 2.1. Materials

Polypropylene (PP, BX3920) with a melt flow index of 100 g/10 min (2.16 kg at 230 °C) was supplied by SK Group (Seoul, Republic of Korea). High-melt-index polypropylene (HPP, MF650X) with a melt flow index of 1200 g/10 min (2.16 kg at 230 °C) was supplied by LyondellBasell Corporation (Jubail, Saudi Arabia). Maleic anhydride-grafted polypropylene (MAPP, Orevac^®^ CA100) was supplied by Arkema (Paris, France). Four commercially available flame retardants (Decabromodiphenyl ethane (DBDPE), halogen-free one-component flame retardant (MONO), halogen-free compound flame retardant (MULTI), and intumescent flame retardant (IFR)), which are commonly used in polypropylene, were supplied by Yantai Xinxiu Corporation (Yantai, China). Direct roving of glass fiber (GF, 4305S) was supplied by the Chongqing Polycomp International Corporation (Chongqing, China).

### 2.2. Preparation of FR/PP

PP, PP-MF650X, MAPP, and FR were poured into the mixer according to the ratios in Table 1, and mixed well. The homogeneous mixture was poured into the twin-screw extruder for pelletizing, in order to mix the flame retardant homogeneously, and the pelletizing was repeated 3 times.

### 2.3. Preparation of FR/GF/PP Laminates

**Figure 1:**
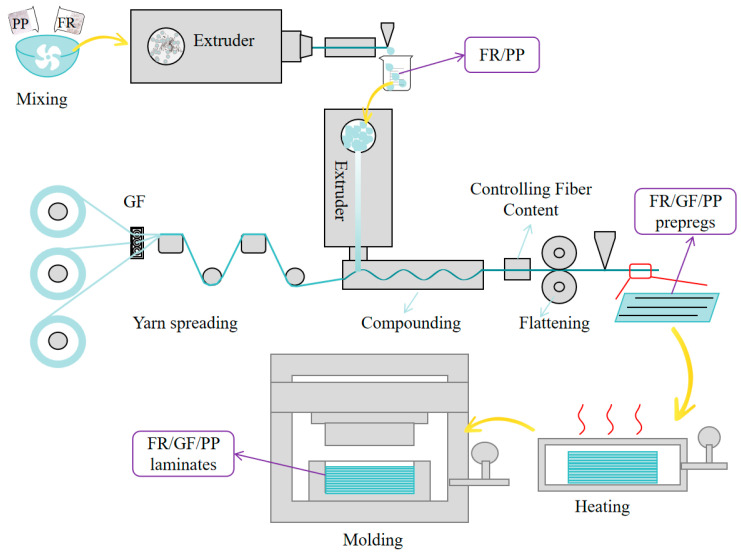
Schematic diagram of preparation process.

A schematic diagram of the preparation process of FR/GF/PP composite laminates is shown in Figure 1. FR/GF/PP prepregs were prepared using a continuous fiber-reinforced thermoplastic composite prepreg production line homemade in the laboratory. FR/PP pellets were melted at the extruder head position and infiltrated with continuous glass fibers, rolled, cooled, and shaped to prepare the FR/GF/PP prepregs. FR/GF/PP prepregs were cut according to the size of the mold; after cutting, the prepreg sheets were placed into the mold to be heated and melted (200 °C, 20 min), then quickly transferred into the molding machine at room temperature for rapid pressurization (25 °C, 7 MPa) and taken out after holding pressure for 10 min. Finally, they were cut into the required sizes for each test using waterjet cutting equipment. In order to reduce the effect of fiber orientation on performance, the FR/GF/PP laminates were unidirectional. The fiber content was 10% volume content.

### 2.4. Characterization

#### 2.4.1. Mechanical Properties

Mechanical property tests were performed on Changchun Kexin WDW-100 Universal Mechanical Testing Machine. According to the ASTM D638 standard [30], the tensile property was tested at the loading speed of 1 mm/min, with a specimen shape of Type I. According to the ASTM D7264 standard [31], a three-point bending performance test was carried out on each specimen at the loading speed of 2 mm/min, with a specimen span-to-thickness ratio of 32.

#### 2.4.2. Dielectric Properties

The dielectric constant and dielectric loss of each specimen at X-band (8.2–12.4 GHz) electromagnetic waves were measured using the rectangular waveguide method on a Model AV3672C Vector Grid Analyzer (China Electronics Technology Group Corporation, Qingdao, China). The rectangular waveguide dielectric constant measurement method utilized the TE mode of electromagnetic waves transmitted from the waveguide to the material to be measured, and the amplitude and phase information of the reflected and transmitted signals were used to invert the dielectric constant of the material to be measured. The specimen size was 10.16 mm × 22.86 mm × 3.0 mm.

#### 2.4.3. Limiting Oxygen Index (LOI)

The Limiting Oxygen Index of each specimen was measured using an Oxygen Indexer (Nanjing Jiangning Analytical Instrument Co., Ltd., Nanjing, China), according to the ASTM D2863 standard [32] test method. The LOI measured the minimum concentration of oxygen required for the flaming combustion of the material in a mixed oxygen-nitrogen gas stream. The LOI specimen size was 6.5 mm × 120 mm × 3.0 mm. The number of parallel specimens was about 15, and the experimental results were statistical results according to ASTM D2863.

#### 2.4.4. Vertical Burning Test

Flame retardant rating was tested using a vertical combustion testing machine (YK-Y0142, YAOKE, Nanjing, China) according to the UL94 flame retardant rating (FRR) test method. The flame retardancy rating was used to evaluate the ability of a material to extinguish after ignition. The FRR specimen size was 13 mm × 120 mm × 3.0 mm. The number of parallel specimens was 10, and the experimental results were statistical results according to UL94.

#### 2.4.5. Scanning Electron Microscope (SEM)

The microscopic morphology of each specimen was observed using a Focused Ion Beam-Scanning Electron Microscope (Helios G4 CX, ThermoFisher Scientific, Brno, Czech Republic). The observation position was the fracture part of the specimen after the bending test. The samples were sprayed gold and tested at an accelerating voltage of 15 kV. The magnification was 2000 times.

## 3. Results and Discussion

### 3.1. Mechanical Properties of GF/PP Modified with Different Flame Retardants

#### 3.1.1. Tensile Properties of PP Modified with Different Flame Retardants

Among all the specimens, the polypropylene specimen without the flame-retardant addition is used as the blank control group. The tensile strength of the control group is 23.04 MPa and the tensile modulus is 1.60 GPa.

**Figure 2:**
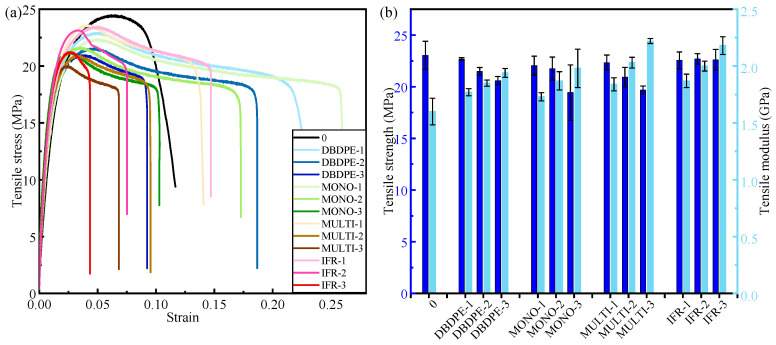
(**a**) Stress–strain curves, (**b**) tensile modulus and tensile strength of PP modified with different flame retardants.

It is obvious from the test results in Figure 2 that the tensile strength and elongation at the break of FR/PP show a decreasing trend with the increase in flame-retardant content, while the tensile modulus increases gradually, whether it is DBDPE, MONO, MULTI or IFR flame retardant. There are differences in the effect of the type of flame retardant on the tensile properties of polypropylene.

When the amount of flame retardant added is lower (8%), the reduction in tensile strength of the four flame retardant-modified PP is not obvious. However, when the flame retardant additive amount is increased to 20.69%, the tensile strengths of MONO and MULTI-modified PP are 19.44 MPa and 19.69 MPa, which are significantly decreased compared to the control group without the flame retardant, which are decreased by 15.63% and 14.54%, respectively. The tensile strengths of DBDPE-modified PP are also decreased with the increase in the content of flame retardant; at flame-retardant additions of 8.00%, 14.81%, and 20.69%, the tensile strengths are 22.69 MPa, 21.48 MPa, and 20.60 MPa, respectively, which are decreased by 1.52%, 6.77%, and 10.59%, respectively, compared to the control specimens with no flame retardant added. IFR-modified PP shows stable tensile strength, and the flame-retardant content has no negative effect on the tensile strength of IFR-modified PP. Among the specimens with IFR as the added flame retardant, the tensile strengths are 22.56 MPa, 22.69 MPa, and 22.61 MPa at the flame-retardant additions of 8.00%, 14.81%, and 20.69%, respectively, and the tensile strengths of the IFR-modified PP show stable performance.

The tensile modulus of the four flame retardant-modified PP increases with the increase in flame-retardant content. The tensile modulus of the polypropylene specimens with DBDPE, MONO, MULTI and IFR flame retardants reaches the maximum value of 1.94 GPa, 1.98 GPa, 2.22 GPa, and 2.18 GPa, respectively, when the flame-retardant content is 20.69%. Among them, the MULTI- and IFR-modified PP have the best effect in improving tensile modulus, which is higher than that of the control group by 38.75% and 36.25%, respectively.

The addition of flame retardants hinders the movement of the chain segments of PP, increases the stiffness, and increases the tensile modulus, but also introduces defects into the matrix, resulting in a decrease in tensile strength due to increased defects. Considering the results of combined tensile strength and tensile modulus, the tensile properties of IFR-modified PP have significant advantages. IFR has better compatibility with PP.

#### 3.1.2. Bending Properties of GF/PP Composites Modified with Different Flame Retardants

**Figure 3:**
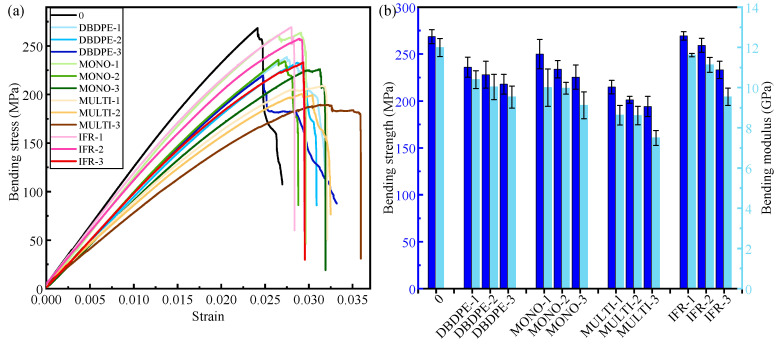
(**a**) Bending stress–strain curves, (**b**) bending modulus and bending strength of PP modified with different flame retardants.

The three-point bending properties of GF/PP composites modified with different flame retardants are presented in Figure 3. The bending strength of the blank control composite is 268.56 MPa and the bending modulus is 11.99 GPa.

The bending strength and bending modulus of each flame retardant-modified GF/PP show a decreasing trend with the increase in flame-retardant addition. Taking IFR/GF/PP as an example, when the flame retardant additive amount is 8.00%, the bending strength and bending modulus of IFR-modified PP are 269.4 MPa and 11.62 GPa, respectively, and the performance is almost unchanged compared with that of the blank control group, and even the average bending strength is slightly increased. When the flame retardant additive amount is increased to 20.69%, the bending strength and bending modulus decrease dramatically to 233.14 MPa and 9.54 GPa, respectively, which are 13.19% and 20.43% lower than the blank control group, respectively.

Comparing the four flame retardants, the bending strength and bending modulus of the MULTI-modified GF/PP drop the most obviously, and the bending strength and bending modulus drop to 194.2 MPa and 7.5 GPa when the flame-retardant addition is increased to 20.69%, which is 27.69% and 37.45% lower than that of the blank control group, respectively. In Section 3.1.1, concerning the MONO and MULTI-modified PP, the tensile properties of the two are similar, but when they modify GF/PP there is a gap and the bending properties of the MULTI-modified GF/PP are significantly lower, which indicates that the MULTI-modified PP has poor composite properties with glass fibers and does not form an effective load transfer interface.

**Figure 4:**
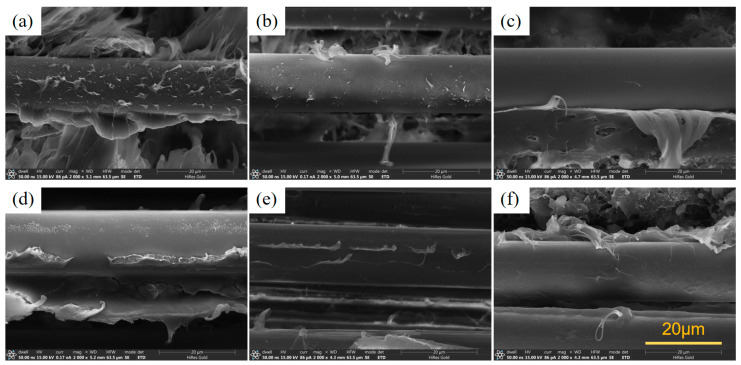
SEM images of GF/PP modified with different flame retardants, (**a**) DBDPE-1, (**b**) MONO-1, (**c**) MULTI-1, (**d**) IFR-1, (**e**) IFR-2, and (**f**) IFR-3.

From Figure 4a–d, it can be seen that DBDPE-, MONO- and IFR-modified PP have a better compounding ability with fibers, and there is still a large amount of resin remaining on the surface of the fibers after they are detached, while MULTI-modified PP has very poor combining ability with fibers; the fiber surface is smooth and almost no MULTI-modified PP remains. From Figure 4d–f, it can be seen that with the increase in the flame-retardant content of the fiber, the surface residue of the resin decreases, and the IFR-modified PP and fiber composite ability decreases.

In Section 3.1.1, the tensile modulus of FR/PP is enhanced with the increase in flame-retardant content, but the phenomenon where the bending modulus increases with the flame retardant does not occur in FR/GF/PP. This is due to the fact that the reinforcing phase in fiber-reinforced composites plays a major role in bearing [33], and the modulus of the matrix and the modulus of the reinforcing body are orders of magnitude different, so the increase in the modulus of the matrix does not play a large role in the overall bending modulus change in the fiber-reinforced composites. While the addition of flame retardant affects the interfacial properties between the matrix and the fiber, the interfacial phase has a particularly important role in the composite material, which is an extremely important microstructure of the composite material, and its structure and properties directly affect the performance of the composite material [34,35]. With the increase in flame-retardant content, the viscosity of matrix melt increases, resulting in a poorer effect of matrix infiltration of fibers, which leads to the interface not being effective in transferring loads and the mechanical properties being reduced. The information presented in Figure 3 shows that the effect of IFR flame retardants on the bending properties of FR/GF/PP is minimized at the same additive level.

### 3.2. Flame-Retardant Properties of GF/PP Composites Modified with Different Flame Retardants

#### 3.2.1. LOI of GF/PP Composites Modified with Different Flame Retardants

**Figure 5:**
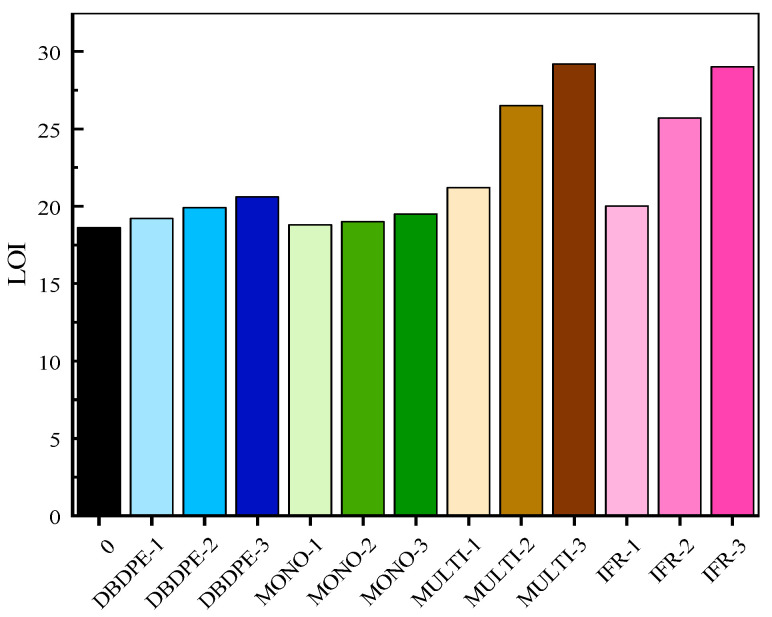
LOI of GF/PP composites modified with different flame retardants.

The LOI data of different flame retardant-modified GF/PP composites are presented in Figure 5, from which it is relatively intuitive to observe that the LOI of flame retardant-modified GF/PP shows an increasing trend with the increase in the flame-retardant content, and the type of flame retardant has a great influence on the LOI of FR/GF/PP.

For the specimens with the addition of DBDPE and MONO flame retardants, the variation in the LOIs is not significant, the enhancement of flame retardant performance is limited, and the flame retardant effect is not ideal. The LOIs of the composite specimens with MULTI and IFR flame retardants increase significantly with the increase in flame-retardant addition. In the samples with MULTI flame retardant, the LOIs of the samples are 21.2, 26.5 and 29.2 at the content of 8.00%, 14.81% and 20.69% of flame retardant, respectively, which are 13.97%, 42.47% and 56.99% higher than that of the samples in the blank control group. In the samples with the IFR flame retardant, the LOIs of the samples are 20.0, 25.7 and 29.0 at the contents of 8.00%, 14.81% and 20.69% of flame retardant, respectively, which are 7.52%, 38.17% and 55.91% higher than those of the samples of the blank control group, respectively. MULTI and IFR are multi-component flame retardants and have higher LOI. Multi-component flame retardants can inhibit material combustion in more ways than a single component, which may result in a better LOI.

#### 3.2.2. Flame Retardant Rating of GF/PP Composites Modified with Different Flame Retardants

**Figure 6:**
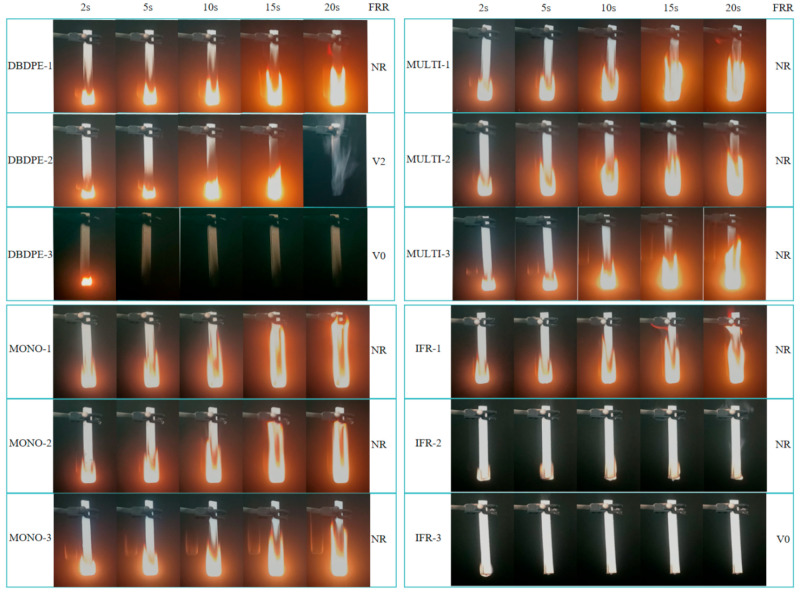
Test photos of vertical burning performance and FRR of GF/PP composites modified with different flame retardants.

The combustion process of the vertical combustion rating test of composite specimens with different flame retardants is presented in Figure 6.

At 8% flame-retardant content, all four types of flame retardant specimens appear to have flame extension and continuous combustion after 10 s of ignition time. The composite specimens with the MONO flame retardant have the fastest burning speed; the flame extended to the top of the specimen after 20 s of withdrawing from the ignition source, while the flame burned to the middle of the specimen for the other three types of specimens.

At 14.81% flame-retardant content, after 10 s of ignition time, the flame of the DBDPE-added specimen had a short extension, then the flame gradually weakened, and the combustion stopped at 20 s; it can be seen in the photo that a large amount of smoke is generated after the combustion has stopped. For the MULTI-added specimen, after the ignition time, the flame continued to expand, the combustion process continued, and after 20 s, the combustion flame was at the middle of the specimen. For the MONO-added specimen, after the fire source withdrew from the specimen, the flame expanded rapidly; during the observation time, the flame did not appear to weaken, and after 20 s the specimen combustion flame reached the specimen clamping position. For the IFR-added specimen, after 10 s of ignition time, the specimen ignited, but the flame was small and the flame expansion was very slow. After 20 s, although the flame was not completely extinguished, it seemed to be very weak.

At 14.81% flame-retardant content, specimens with DBDPE and IFR additions show a brief 1–3 s of burning after the ignition time, followed by immediate self-extinguishing. The specimens with the MULTI and MONO additions continue to burn after the ignition time, with a significant expansion of the flame, and are still not extinguished after 20 s.

Among the four kinds of flame retardants, only the DBDPE-1 and DBDPE-2 specimens are accompanied with the phenomenon of resin molten droplets igniting the bottom skimming cotton during combustion. Finally, the FRRs are obtained for DBDPE-2-, DBDPE-3- and IFR-3-modified GF/PP specimens, and their FRRs are V2, V0 and V0, respectively.

### 3.3. Dielectric Properties of GF/PP Modified with Different Flame Retardants

The relation between the dielectric constant and the angle *θ* of the continuous fiber-reinforced composite [36] is shown in Equation (4). This rule does not change with the variation in flame-retardant content.

(1) $$\varepsilon_{11}^{\prime }=\varepsilon_{f}^{\prime }\upsilon_{f}+\varepsilon_{m}^{\prime }\upsilon_{m}$$

(2) $$\varepsilon_{22}^{\prime }=\frac{\varepsilon_{m}^{\prime 2}+\varepsilon_{m}^{\prime }\sqrt{\upsilon_{f}}\left({\bar{\varepsilon }}_{f2}^{\prime }-\varepsilon_{m}^{\prime }\right)}{\varepsilon_{m}^{\prime }\left(1+\upsilon_{f}-\sqrt{\upsilon_{f}}\right)+{\bar{\varepsilon }}_{f2}^{\prime }\left(\sqrt{\upsilon_{f}}-\upsilon_{f}\right)}$$

where

(3) $${\bar{\varepsilon }}_{f2}^{\prime }=\varepsilon_{m}^{\prime }+\frac{\pi }{4}\left(\varepsilon_{f}^{\prime }-\varepsilon_{m}^{\prime }\right)$$

(4) $${\left[\varepsilon_{ij}^{\prime }\right]}^{\theta }=\varepsilon_{11}^{\prime }{cos}^{2}\theta +\varepsilon_{22}^{\prime }{sin}^{2}\theta$$

where $\varepsilon_{11}^{\prime }$ and $\varepsilon_{22}^{\prime }$ are the dielectric constants at angles of 0 and 90, respectively. $\varepsilon_{f}^{\prime }$ and $\varepsilon_{m}^{\prime }$ are the dielectric constants of the fiber and matrix, respectively. $\upsilon_{f}$ and $\upsilon_{m}$ are the volume contents of the fiber and matrix, respectively, and ${\left[\varepsilon_{ij}^{\prime }\right]}^{\theta }$ is the dielectric constant when the angle is θ.

**Figure 7:**
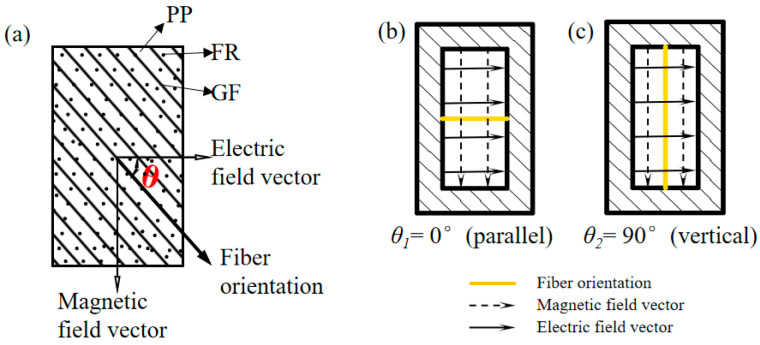
Schematic diagram of fiber direction and electric field direction, (**a**) *θ* position, (**b**) *θ* = 0°, (**c**) *θ* = 90°.

**Figure 8:**
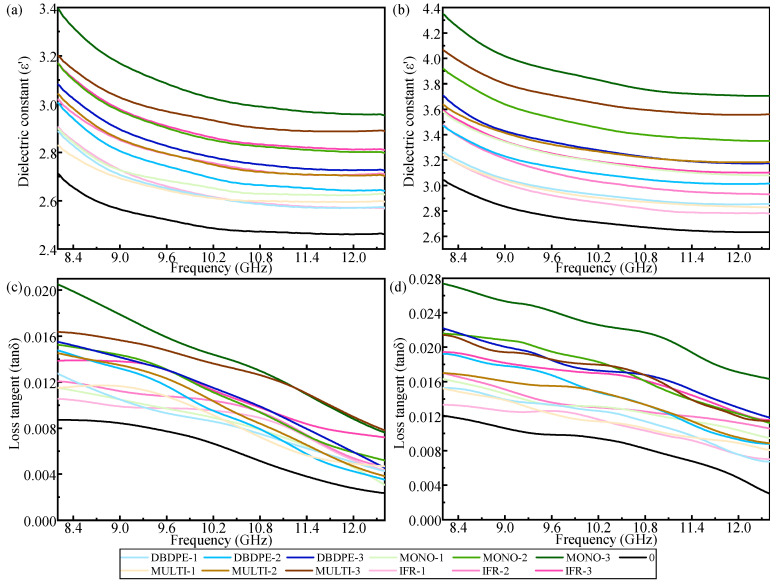
Dielectric properties versus frequency of FR/GF/PP samples, (**a**) dielectric constant at *θ* = 90°, (**b**) dielectric constant at *θ* = 0°, (**c**) dielectric loss tangent at *θ* = 90°, and (**d**) dielectric loss tangent at *θ* = 0°.

**Figure 9:**
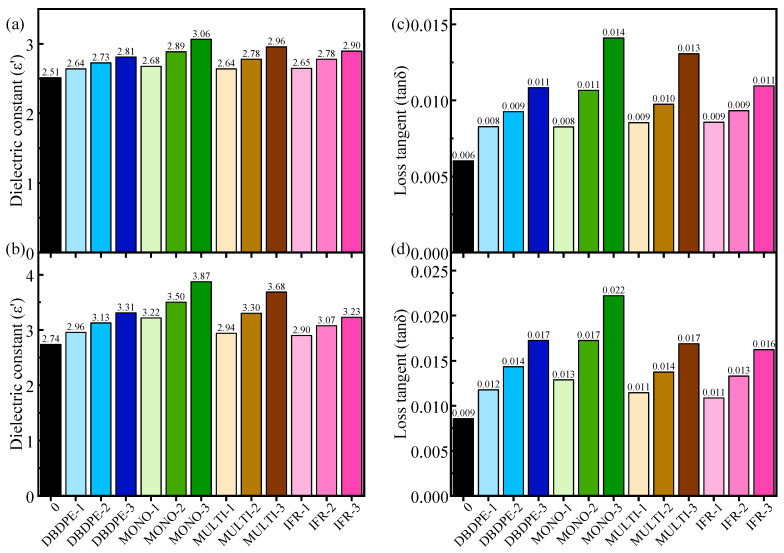
Average dielectric properties of FR/GF/PP samples in X-band, (**a**) dielectric constant at *θ* = 90°, (**b**) dielectric constant at *θ* = 0°, (**c**) dielectric loss tangent at *θ* = 90°, and (**d**) dielectric loss tangent at *θ* = 0°.

It can be concluded from Equation (4) that the dielectric properties of unidirectional continuous fiber composite panels decrease with the increase in the angle *θ* (Figure 7) between the fibers and the direction of the electric field, which is at its maximum at *θ* = 0° (parallel) and minimum at *θ* = 90° (vertical). So in this paper, the dielectric properties are tested for both *θ* = 0° and *θ* = 90° directions. The dielectric properties in each direction are shown in Figure 8 and Figure 9.

The dielectric constant and dielectric loss tangent of FR/GF/PP increase with the increase in flame-retardant content when the flame retardant type is the same. It can be hypothesized from this result that probably the dielectric properties of the flame retardant are higher than those of the PP matrix. As the flame-retardant content increases, the interfacial polarization between the resin and fibers is also enhanced, resulting in an increase in the total dielectric constant and dielectric loss tangent of the composite. The addition of large amounts of flame retardants has a very significant effect on the dielectric properties, especially the dielectric loss values, so it is necessary to reduce the flame-retardant content to maintain the low dielectric properties of polypropylene.

At the same flame-retardant content, the dielectric properties of different kinds of flame retardants have different extents of increase. When the flame-retardant content is increased to 20.69%, the parallel dielectric constants of GF/PP modified with four kinds of flame retardants (DBDPE, MONO, MULTI, IFR) increase to 3.31, 3.87, 3.68 and 3.23, respectively, which are 20.87%, 41.32%, 34.53% and 17.88% more than those of the blank group, and the vertical dielectric constants increase to 2.81, 3.06, 2.96 and 2.90, which are 11.88%, 21.92%, 17.70% and 15.23% more than those of the blank group, respectively.

When the flame-retardant content is increased to 20.69%, the parallel dielectric loss tangents of GF/PP modified with four kinds of flame retardants (DBDPE, MONO, MULTI, IFR) increase to 0.01723, 0.02219, 0.01686 and 0.01621, respectively, which are 101.29%, 159.23%, 96.96% and 89.37% more than those of the blank group, and the vertical dielectric loss tangents increase to 0.01083, 0.0141, 0.01307 and 0.01095, which are 79.60%, 133.83%, 116.75% and 81.59% more than those of the blank group, respectively.

Among the four different types of FR/GF/PP, DBDPE- and IFR-modified GF/PP have lower dielectric constants and dielectric loss tangents at the same flame-retardant content. This can be attributed to the fact that these two flame retardants may have lower polarizabilities themselves, and lower interfacial polarizations because of better interfaces (Figure 4).

The dielectric constant and dielectric loss tangent of each specimen show a decreasing trend with the increase in frequency, and this trend does not change with the addition of flame retardant type, which is due to the time needed for the polarization of the dielectric and because the polarization time of various polarization methods is different. With the frequency increase, the electric field change period becomes shorter, the internal polarization of the material gradually lags behind the electric field change, and part of the polarization will not work, so the dielectric properties are reduced.

### 3.4. Comprehensive Performance Evaluation

The above analysis can be used to briefly analyze the effects of flame retardant type and flame-retardant content on bending properties, flame-retardant properties and dielectric properties, respectively; for example, the increase in flame-retardant content is helpful for the improvement of flame retardant performance, but a large addition will reduce the mechanical properties and increase the dielectric properties. There is no one flame retardant that can obtain the optimal performance in all the properties; for example, IFR has the highest performance in mechanical properties, MULTI has a better performance in LOI, DBDPE and IFR have better performances in FRR, and DBDPE and IFR have lower and similar dielectric properties. Therefore, certain statistical rules are needed to select the most suitable flame retardant type and flame-retardant content value for the application in this paper from many combinations.

In order to obtain the optimal overall performance, gray relational analysis is applied to deal with the data of each specimen. Gray relational analysis can transform the multi-objective multi-response problem into a single gray relational comparison problem. The comprehensive performance of each specimen is compared by the final gray relational comparison.

The experiment data of each test are summarized in Table 2. In order to facilitate data processing, the flame retardant level V0 is assigned to 3, V1 is assigned to 2, V2 is assigned to 1, and no grade is assigned to 0. The dielectric constant and dielectric loss tangent are the average of the X-band test values in both directions.

In order to eliminate the effect of magnitude, the data obtained from the experiments are homogenized. Among the objectives, bending strength, bending modulus, LOI and FRR are the larger-the-better objectives, which are homogenized using Equation (5); the dielectric constant and dielectric loss are the smaller-the-better objectives, which are processed using Equation (6). The values of each objective after homogenization are summarized in Table 3.

(5) $$x_{i}\left(k\right)=\frac{y_{i}(k)-\min y_{i}(k)}{\max y_{i}(k)-\min y_{i}(k)}$$

(6) $$x_{i}^{\prime }\left(k\right)=\frac{\max y_{i}(k)-y_{i}(k)}{\max y_{i}(k)-\min y_{i}(k)}$$

The homogenized data are processed using Equation (7) to obtain the gray correlation coefficient of each specimen in each performance. The gray correlation coefficient can reflect the degree of correlation between the actual value and the expected value, and the larger the gray correlation coefficient is, the closer it is to the optimization goal.

(7) $$\xi_{i}(k)=\frac{\Delta \min+\zeta \times \Delta \max}{\left|x_{0}(k)-x_{i}(k)\right|+\zeta \times \Delta \max}$$

where

(8) $$\Delta \min=\underset{i}{\min}\underset{k}{\min}\left|x_{0}(k)-x_{i}(k)\right|$$

(9) $$\Delta \max=\underset{i}{\max}\underset{k}{\max}\left|x_{0}(k)-x_{i}(k)\right|$$

*x*_0_(*k*) is the reference data column and refers to the maximum value of each performance index after homogenization: here *x*_0_(*k*) = 1. ζ is the resolution coefficient, $\zeta \in \left[0,1\right]$, and in this paper takes 0.5. $\xi_{i}(k)$ is called the correlation coefficient of *x_i_* to *x*_0_ with respect to the *k* indicator. The gray correlation coefficient of each performance is summarized in Table 4.

The gray correlation for each specimen is the sum of the multiples of each performance weight and the corresponding gray correlation coefficient, which is calculated using Equation (10). *R_i_* is the gray correlation of the *i*th specimen.

(10) $$R_{i}=\sum_{k=1}^{n}\omega (k)\xi_{i}(k)$$

The evaluation of the weights for each objective is quite subjective, and different workers will have different weights assigned for different applications. The weights of each performance in this paper are obtained using the analytic hierarchy process (AHP) according to the set application. In the application scenario of this paper, the flame retardant performance is required to be the highest, which is required to reach V0 grade, followed by dielectric properties, which are required to be as low as possible. The bending properties (bending strength greater than 200 MPa, bending modulus greater than 9 GPa) can be achieved in most of the specimens; therefore, flame-retardant properties are considered the most important, followed by dielectric properties, and finally, mechanical properties.

AHP calculates weight vectors through building hierarchical models and constructing judgment matrices [37,38,39]. The hierarchical model designed is presented in Table 5.

The judgment matrix of the analytic hierarchy process is presented in Table 6.

By using the judgment matrix, the weights of each performance are calculated according to Equation (11).

(11) $$\omega_{i}=\frac{b_{i}}{\sum_{k=1}^{n}b_{k}}$$

where

(12) $$b_{i}={\left(\prod_{j=1}^{n}a_{ij}\right)}^{\frac{1}{n}}$$

*n* is the number of indicator factors in each layer; *a_ij_* is the relative importance of indicator *i* compared to factor *j*. The values of *a_ij_* are within the judgment matrix (Table 6), with 1 indicating that factors *i* and *j* are equally important, 3 indicating that *i* is slightly more important than factor *j*, and 5 indicating that *i* is significantly more important than factor *j*.

Each performance of Layer C is considered equally important relative to Layer B. The final weights of each layer are presented in Table 7.

The weights from Table 8 and the gray correlation coefficients from Table 4 are brought into Equation (10) to calculate the gray correlation for each specimen.

As shown in Table 8, IFR-3 has the highest gray correlation value, and the excellent flame retardant performance of the IFR flame retardant has greatly improved its gray correlation value. IFR has a better compounding ability with GF/PP and has higher mechanical properties and lower dielectric properties. The second is DBDPE-3, which is better in flame retardancy; the GF/PP with DBDPE flame retardant has the lowest dielectric properties, but its mechanical properties are poor, resulting in the overall evaluation of the second to IFR. Due to the addition of flame retardants, in the other combinations the mechanical properties deteriorate, the dielectric properties increase and the flame retardant performance is mediocre, resulting in gray correlation values even lower than the blank group of specimens. Therefore, in the application scenario set in this paper, the IFR flame retardant with 20.69% flame-retardant content is selected. Gray relational analysis is generally used for process parameter optimization, but in this paper it is applied for formulation selection. It provides ideas for multi-objective multi-response material formulation selection.

## 4. Conclusions

When the flame retardant types are the same, as the most important role of adding flame retardants, the flame-retardant properties of FR/GF/PP increase with the increase in flame-retardant content. However, with the increase in flame-retardant content, the mechanical properties of FR/GF/PP will be reduced and the dielectric properties will be elevated.

In addition to the flame-retardant content, the flame retardant type also has a significant effect on the properties of flame retardant-modified GF/PP. The effect of the flame retardant type on various properties is inconsistent. This is determined by their respective elemental composition and structure. Among the four flame retardant-modified GF/PP, IFR has the highest performance in mechanical properties, MULTI has a better performance in LOI, DBDPE and IFR have better performances in FRR, and DBDPE and IFR have lower and similar dielectric properties. Thus, it is necessary to find the most suitable flame retardant type and flame-retardant content value for the application scenario through certain statistical rules.

In this paper, gray relational analysis is used for formulation selection. The analytic hierarchy process is used to assign the weights of each performance target in combination with the set application occasions, and finally, the flame retardant type and flame-retardant content that optimize the comprehensive performance of FR/GF/PP, namely IFR-3, are obtained.

## Figures and Tables

**Table 1:** Designation and composition of FR/PP.

Samples	PP (phr)	MAPP (phr)	HPP (phr)	FR (phr)	FR Content (wt%)
0	100	5	10	0	0
X-1	100	5	10	10	8.00
X-2	100	5	10	20	14.81
X-3	100	5	10	30	20.69

Where X is flame retardant (DBDPE, MONO, MULTI, IFR) and flame-retardant content = flame retardant/(PP + MAPP + HPP + flame retardant).

**Table 2:** Experiment data of each sample.

Samples	Bending Strength (MPa)	Bending Modulus (GPa)	LOI (%)	FRR	Dielectric Constant	Dielectric Loss Tangent
0	268.56	11.99	18.6	0	2.6258	0.0073
DBDPE-1	236.00	10.39	19.2	0	2.7980	0.0100
DBDPE-2	228.09	10.03	19.9	1	2.9262	0.0118
DBDPE-3	217.84	9.53	20.6	3	3.0609	0.0140
MONO-1	249.98	10.00	18.8	0	2.9475	0.0106
MONO-2	233.88	9.96	19	0	3.1938	0.0139
MONO-3	225.56	9.12	19.5	0	3.4670	0.0181
MULTI-1	214.79	8.63	21.2	0	2.7896	0.0100
MULTI-2	201.35	8.61	26.5	0	3.0401	0.0117
MULTI-3	194.19	7.50	29.2	0	3.3210	0.0150
IFR-1	269.40	11.62	20	0	2.7740	0.0097
IFR-2	259.37	11.13	25.7	0	2.9272	0.0113
IFR-3	233.14	9.54	29	3	3.0620	0.0136

**Table 3:** Homogenization date of each sample.

Samples	Bending Strength	Bending Modulus	LOI	FRR	Dielectric Constant	Dielectric Loss Tangent
0	0.99	1	0	0	1	1
DBDPE-1	0.56	0.64	0.06	0	0.80	0.75
DBDPE-2	0.45	0.56	0.12	0.33	0.64	0.59
DBDPE-3	0.31	0.45	0.19	1	0.48	0.38
MONO-1	0.74	0.56	0.02	0	0.62	0.70
MONO-2	0.53	0.55	0.04	0	0.32	0.39
MONO-3	0.42	0.36	0.08	0	0	0
MULTI-1	0.27	0.25	0.25	0	0.81	0.75
MULTI-2	0.10	0.25	0.75	0	0.51	0.59
MULTI-3	0	0	1	0	0.17	0.29
IFR-1	1	0.92	0.13	0	0.82	0.78
IFR-2	0.87	0.81	0.67	0	0.64	0.63
IFR-3	0.52	0.45	0.98	1	0.48	0.42

**Table 4:** Gray correlation coefficient of each sample.

Samples	Bending Strength	Bending Modulus	LOI	FRR	Dielectric Constant	Dielectric Loss Tangent
0	0.98	1.00	0.33	0.33	1.00	1.00
DBDPE-1	0.53	0.58	0.35	0.33	0.71	0.67
DBDPE-2	0.48	0.53	0.36	0.43	0.58	0.55
DBDPE-3	0.42	0.48	0.38	1.00	0.49	0.45
MONO-1	0.66	0.53	0.34	0.33	0.57	0.62
MONO-2	0.51	0.53	0.34	0.33	0.43	0.45
MONO-3	0.46	0.44	0.35	0.33	0.33	0.33
MULTI-1	0.41	0.40	0.40	0.33	0.72	0.67
MULTI-2	0.36	0.40	0.66	0.33	0.50	0.55
MULTI-3	0.33	0.33	1.00	0.33	0.38	0.41
IFR-1	1.00	0.86	0.37	0.33	0.74	0.69
IFR-2	0.79	0.72	0.60	0.33	0.58	0.58
IFR-3	0.51	0.48	0.96	1.00	0.49	0.46

**Table 5:** Hierarchical model.

A	B	C
Comprehensive performance (A1)	Flame-retardant properties (B1)	LOI (C1)
		FRR (C2)
	Dielectric properties (B2)	Dielectric constant (C3)
		Dielectric loss tangent (C4)
	Mechanical properties (B3)	Bending strength (C5)
		Bending modulus (C6)

**Table 6:** Judgment matrix.

A1	B1	B2	B3
B1	1	3	5
B2	1/3	1	3
B3	1/5	1/3	1

**Table 7:** Weights of each layer.

B	B~A	C	C~B	C~A
Flame-retardant properties (B1)	0.6370	LOI (C1)	0.5	0.3185
		FRR (C2)	0.5	0.3185
Dielectric properties (B2)	0.2583	Dielectric constant (C3)	0.5	0.1291
		Dielectric loss tangent (C4)	0.5	0.1291
Mechanical properties (B3)	0.1047	Bending strength (C5)	0.5	0.0524
		Bending modulus (C6)	0.5	0.0524

**Table 8:** Gray correlation values for each specimen.

**Samples**	**0**	**DBDPE-1**	**DBDPE-2**	**DBDPE-3**	**MONO-1**	**MONO-2**	**MONO-3**
Gray correlation	0.57	0.45	0.45	0.61	0.43	0.38	0.35
**Samples**	**MULTI-1**	**MULTI-2**	**MULTI-3**	**IFR-1**	**IFR-2**	**IFR-3**	
Gray correlation	0.45	0.49	0.56	0.5	0.53	0.8	

## Data Availability

Data are contained within the article.
